# Investigation of coagulation and proteomics profiles in symptomatic feline hypertrophic cardiomyopathy and healthy control cats

**DOI:** 10.1186/s12917-024-04170-0

**Published:** 2024-07-05

**Authors:** Palin Jiwaganont, Sittiruk Roytrakul, Siriwan Thaisakun, Pratch Sukumolanan, Soontaree Petchdee

**Affiliations:** 1 Graduate School, Veterinary Clinical Studies Program, Faculty of Veterinary Medicine, Kasetsart University, Kamphaeng Saen, Nakorn Pathom, Thailand; 2 Functional Proteomics Technology Laboratory, National Center for Genetic Engineering and Biotechnology (BIOTEC), National Science and Technology Development Agency, Pathum Thani, Thailand; 3 Department of Large Animal and Wildlife Clinical Sciences, Faculty of Veterinary Medicine, Kasetsart University, Kamphaeng Saen, Nakorn Pathom, Thailand

**Keywords:** D-dimer, Prothrombin time, Proteomics, Hypertrophic cardiomyopathy, Cats

## Abstract

**Background:**

Hypertrophic cardiomyopathy (HCM) is a crucial heart disease in cats. The clinical manifestations of HCM comprise pulmonary edema, dyspnea, syncope, arterial thromboembolism (ATE), and sudden cardiac death. D-dimer and prothrombin time (PT) are powerful biomarkers used to assess coagulation function. Dysregulation in these two biomarkers may be associated with HCM in cats. This study aims to assess D-dimer levels, PT, and proteomic profiling in healthy cats in comparison to cats with symptomatic HCM.

**Results:**

Twenty-nine client-owned cats with HCM were enrolled, including 15 healthy control and 14 symptomatic HCM cats. The D-dimer concentration and PT were examined. Proteomic analysis was conducted by matrix-assisted laser desorption ionization time-of-flight (MALDI-TOF) mass spectrometry and liquid chromatography-tandem mass spectrometry (LC-MS/MS). In symptomatic cats, D-dimer levels were statistically significantly higher (mean ± SEM: 372.19 ng/ml ± 58.28) than in healthy cats (mean ± SEM: 208.54 ng/ml ± 10.92) with P-value of less than 0.01, while PT was statistically significantly lower in symptomatic cats (mean ± SEM: 9.8 s ± 0.15) compared to healthy cats (mean ± SEM: 11.08 s ± 0.23) with P-value of less than 0.0001. The proteomics analysis revealed upregulation of integrin subunit alpha M (ITGAM), elongin B (ELOB), and fibrillin 2 (FBN2) and downregulation of zinc finger protein 316 (ZNF316) and ectonucleoside triphosphate diphosphohydrolase 8 (ENTPD8) in symptomatic HCM cats. In addition, protein-drug interaction analysis identified the Ras signaling pathway and PI3K-Akt signaling pathway.

**Conclusions:**

Cats with symptomatic HCM have higher D-dimer and lower PT than healthy cats. Proteomic profiles may be used as potential biomarkers for the detection and management of HCM in cats. The use of D-dimer as a biomarker for HCM detection and the use of proteomic profiling for a better understanding of disease mechanisms remain to be further studied in cats.

## Background

Hypertrophic cardiomyopathy (HCM) is a primary heart disease of the myocardium in cats. This disease causes impairment of the diastolic function of the heart. Moreover, it has been reported that feline HCM presents with left ventricular concentric hypertrophy accompanied by enlargement of cardiomyocytes, myocardial disarray, and cardiac fibrosis [1]. The prevalence of HCM in cats is approximately 15% [2]. The current challenge in diagnosing moderate feline HCM lies in the requirement for skilled sonographers. However, even proficient sonographers may encounter disagreements in certain cases due to the variability in measurements and differences between individuals in echocardiography. Distinguishing cats with mild HCM from normal cats can be challenging without employing specific techniques. Some cats exhibit normal left atrium size, no systolic anterior motion (SAM), large papillary muscles, and borderline left ventricular wall thickness, falling into the category of an equivocal phenotype, and these cats may or may not develop an HCM phenotype qa [3]. Transthoracic echocardiography to measure left ventricular wall thickness is considered the gold standard for diagnosis of HCM and classifying this disease into four stages (stage A, B1, B2, C, and D) as recommended in the American College of Veterinary Internal Medicine (ACVIM) guidelines [4]. Due to the various stages of HCM in cats, numerous cats with HCM do not exhibit clinical or asymptomatic signs, accounting for 46.5%. However, approximately 53.5% of HCM-affected cats have demonstrated common clinical manifestations, including cardiogenic pulmonary edema, pleural effusion, syncope, hypothermia, arterial thromboembolism (ATE), and sudden cardiac death [5, 6].

Thromboembolism can obstruct distant arterial vessels, such as femoral and brachial arteries. The pathophysiology of ATE is still unknown [7]. However, ATE is strongly associated with feline HCM through hypokinesia and left atrial (LA) chamber enlargement. Moreover, it has been reported that cats with ATE and congestive heart failure due to HCM are at a higher risk of death [8]. Therefore, proper management and early detection are essential to prevent a worst-case scenario of this disease in cats.

As part of Virchow’s triad, hypercoagulation is one of the critical factors for thrombogenesis in the cardiac chamber. Consequently, little evidence shows that evaluating the coagulation profile would enhance patient outcomes. Assessing the coagulation profile is necessary for gaining a better understanding of coagulation parameters in cats with HCM. This would not only improve our understanding of the pathogenesis but also potentially aid in the development of future drugs for this disease. D-dimer, a powerful biomarker for blood clotting, is released during both blood clot formation and breakdown [9]. PT is used to identify abnormalities in the extrinsic and common coagulation pathways [10]. Abnormalities in these two coagulation markers are closely linked to various coagulation disorders, such as disseminated intravascular coagulation (DIC), hepatic cirrhosis, and deep-vein thrombosis [11, 12].

Proteomics is a rapid and influential approach for investigating all proteins expressed in specific tissues, whole blood, serum, or urine. It is valuable for learning pathophysiology, exploring novel biomarkers, or monitoring disease progression. Various publications have utilized proteomics analysis to better understand feline HCM with multiple conditions, such as Maine Coon cats with sarcomeric gene mutations and Bengal cats with *MYBPC3*-A74T mutations [13, 14].

A previous proteomics study was conducted in cats with congestive heart failure due to primary cardiomyopathy. The proteins, such as serine protease inhibitors (SERPINs), prothrombin, thymosin β-4 (TMSB4X), antithrombin III (ATIII), α-2 antiplasmin (A2AP), were reported to increase in the cardiomyopathy cats. However, several immunoglobin protein concentrations were significantly lower in the cardiomyopathy cats [15]. Recently, proteomics research has been rising in veterinary medicine. However, there are only a few reports on using proteomics technology to study cat cardiomyopathy.

We hypothesized that proteomics analysis would distinguish the proteins between symptomatic and healthy control cats and identify potential biomarkers for symptomatic HCM. Therefore, this study aims to compare the differences in coagulation markers between healthy cats and HCM stages B2 and C and to determine differences in proteomics profile between a healthy and symptomatic HCM.

## Materials and methods

### Animals and coagulation markers measurement

A cross-sectional investigation with convenience sampling was utilized. This study was conducted at the Kasetsart University School of Veterinary Medicine with IACUC approval of the Kasetsart University (ACKU-62-VET-059). Written informed client consent to participate was obtained from the owners of each enrolled patient in this veterinary clinical trial. All methods were performed in accordance with the relevant ethical guidelines and regulations. This study enrolled twenty-nine owned cats, composed of fifteen healthy cats and fourteen symptomatic cats. Clinical information on recruited cats was obtained, including sex, breed, body weight, and age. The 2 ml of blood samples were collected from venous vessels such as cephalic or medial saphenous veins on the first date of diagnosis. Cats with significant blood profile abnormalities such as anemia, thrombocytopenia, leukopenia, chronic kidney disease, or previous treatment with anticoagulants were excluded from the study. Cats receiving anticoagulants or cats with endocrinopathies, neoplasia, hyperthyroidism, systemic hypertension, and aortic stenosis were excluded from this study. The whole blood from the EDTA tube was kept for genotyping the MYBPC3 gene mutation status at -20 °C until it was analyzed. Moreover, the blood was collected in a tube containing sodium citrate for coagulation markers evaluation. The collected blood was immediately examined for the D-dimer level and PT. D-dimer was assessed by Abbott D-dimer assay applied on the Alinity c clinical chemistry analyzer (Abbott Laboratories, Chicago, IL). Prothrombin time (PT) using two different point-of-care analyzers (Idexx Coag DX and MS Quick Vet Coag Combo).

### Echocardiography

Echocardiography was completed using a board-certified veterinary cardiologist (Asian College of Veterinary Internal Medicine). In this study, the morphology and function of the hearts were determined by a Vivid 5s cardiac ultrasound machine (GE, Boston, MA, USA). Images were obtained with a 6 MHz or 10 MHz transducer. The echocardiographic parameters, including the diameter in both diastole and systole phase of the interventricular septum (IVS), left ventricular internal dimension (LVID), left ventricular proximal wall (LVPW), and percentage of left ventricular fractional shortening (FS), were examined using M-mode echocardiography in the right parasternal short axis at papillary muscle view. Swedish’s method evaluated the left atrial diameter (LA), aorta diameter (AO), and LA/AO ratio [16]. Additionally, the Doppler echocardiography was completed to investigate transmitral E wave velocity (MV E vel), transmitral A wave velocity (MV A vel), and the proportion of MV E vel and MV A vel (E/A ratio). Isovolumic relaxation time (IVRT) was executed to determine the diastolic function with tissue Doppler imaging.

According to the American Heart Association (AHA) and American College of Veterinary Internal Medicine (ACVIM) staging of HCM. Cats in the control group are cats that are classified as stage A, which includes cats in breeds predisposed that may be prone to disease, but there is no evidence of cardiomyopathy. Stage B includes cats with cardiomyopathy or cats with a genetic mutation of *MYBPC3* but no clinical symptoms. Atrial size and thrombus in the left atrium were used to subdivide cats into stages B1 and B2. Stage B1: cats at low risk of congestive heart failure (CHF) or arterial thrombosis (ATE) but have septal and/or LV free (post) wall thickening and stage B2: cats at high risk of developing CHF or ATE. Other factors such as severe LV hypertrophy and cats with symptoms of CHF or ATE are classified as stage C, while cats with CHF resistant to treatment are classified as stage D.

In the present study, echocardiography of left ventricular wall thickness was performed to classify HCM into four stages (stages A, B1, B2, C, and D) as recommended in the ACVIM guidelines [4]. The criteria for diagnosing HCM such as the presence of murmur or arrhythmias on the physical examination or presence in an echo examination based on various parameters, including the dilation of the left atrial (LA) size, left ventricular hypertrophy, with at least left ventricular septal thickening or an interventricular septum measuring greater than 6 mm at end-diastole as seen in M-mode images, spontaneous echo contrast and restrictive diastolic pattern.

However, for this present study, we compared healthy control cats with symptomatic cats stages B1, B2, and C.

### Genotyping of *MYBPC3* gene mutation

The DNA was extracted using the Blood Genomic DNA Extraction Mini Kit (Favorgen, Taiwan), For identification of *MYBPC3:A31P and A74T* gene mutation, the primer was obtained from Godiksen [17]. The protocol for PCR included initial heat at 95 °C for 15 min, followed by 35 cycles of denaturation (95 °C for 30 s), annealing (58 °C for 30 s), and extension (72 °C for 1 min). Finally, the final extension was performed at 72 °C for 10 min. Then, the PCR product was purified according to the manufacturer’s recommendation (Favorgen, Taiwan). After that, the purified product was nucleotide analyzed with the Sanger method. Lastly, the sequencing results were accomplished by using the Bioedit program.

### Proteomics analysis of MALDI-TOF mass spectrometry

Lowry’s method was conducted for the measurement of protein concentration. The protein concentration was detected in the absorbance at 750 nm. The standard curve was plotted compared to the standard protein, bovine serum albumin (BSA) [18]. Briefly, 0.1% trifluoroacetic acid was utilized for acidifying peptide serum to a final concentration of 0.1 mg/ml. Then, one µl peptide was mixed with MALDI solution and spotted with eight replications on the MALDI target (MTP 384 ground steel, Bruker Daltonik, GmbH). After the MALDI-Spiral TOF experiment, all mass spectra were analyzed with flexAnalysis version 3.3 and ClinPro Tools version 3.0 software (Bruker Daltonics, Bremen, Germany), including peptide mass fingerprints (PMFs), pseudo-gel view, and 3-dimensional principal component analysis (3-PCA). Moreover, ProteoMass MALDI Calibration Kits (Sigma Aldrich, St. Louis, Missouri, USA), including ACTH fragment 18–39 (human), insulin oxidized B chain (bovine), insulin (bovine), cytochrome C (equine), and apomyoglobin (equine), were determined as external protein calibration.

### Proteomics analysis of LC-tandem mass spectrometry

This study determined the peptides using a nano-liquid chromatography-electrospray ionization MS/MS analysis with reversed-phase high-performance liquid chromatography (HPLC) to separate the peptide molecules. In brief, the serum was separated using a PepSwift Monolithic Nano Column. Then, the peptides were ionized into gas-phase ions with an electrospray ionization (ESI) system. The peptides were analyzed utilizing an UltiMate 3000 LC System (Thermo Fisher Scientific, Waltham, MA) combined with a PTM Discovery System (Bruker Daltonics). Moreover, a quadrupole ion-trap mass spectrometer (Bruker Daltonics) was conducted for peptide analysis. This analytical technique provided for further protein identification and measurement of the quantity of analytical proteins. The Peptide identification was indicated with the Uniprot program employed to explore the database using the previously analyzed data acquired from the NCBI database [19]. STITCH database (version 5) was used to analyze functional interaction networks between identified proteins from LC-MS data [20]. A list of drugs commonly used to treat cardiac disease, including pimobendan, losartan, telmisartan, furosemide, spironolactone, enalapril, ramipril, benazepril, atenolol, propranolol, amlodipine, aspirin, and clopidogrel, were added to the program to evaluate functional network interactions with the identified proteins.

### Statistical analysis

In this study, all data were represented in mean ± standard deviation (SD). However, D-dimer and prothrombin time data were represented in mean ± standard error of the mean (SEM). Normal distribution was calculated using the Shapiro–Wilk test. Age between groups organized the data from the lowest value to the highest value (median). The paired t-test and chi-square were performed to determine the difference between the healthy and symptomatic HCM cats. For statistical analysis, GraphPad Prism 9 software was performed. A P-value less than 0.05 was a statistically significant difference. Moreover, The LC-MS data was analyzed using Metaboanalyst 5.0 program for visualization and statistical analysis. The analysis included Partial least squares discriminant analysis (PLS-DA) and differential analysis (volcano plot and heatmap) with a significance threshold of P-value < 0.05 [21].

## Results

### Animals and genotypic results

Twenty-nine cats (average aged 3.31 ± 2.54 years and weighing 4.16 ± 0.99 kg) were enrolled in this study, comprising fifteen healthy and fourteen symptomatic cats. Male cats were demonstrated to have greater proportions of healthy and symptomatic HCM, accounting for 60.0% and 54.3%, respectively. The median age was 2.5 years in healthy cats and 2.0 years in symptomatic cats. The cats in the healthy control group were older than those in the symptomatic group However, there were no significant differences in age between cats in the healthy control group and the symptomatic group.

Moreover, the median weight was 4.2 and 3.6 kg in healthy and symptomatic HCM cats, respectively. Table 1 illustrates the clinical information of the enrolled cats. In clinical HCM cats, the clinical presentations included dyspnea, cardiogenic pulmonary edema, and arterial thrombosis. Our study provided results of sarcomeric protein mutations in the *MYBPC3* gene. The genotypic results demonstrated that the mutation rate of all enrolled cats was 31.03%, as displayed in Table 1.

**Table 1:** Clinical information and coagulation markers in enrolled cats

Parameters	Healthy control cats (*n* = 15)	Symptomatic cats (*n*-14)	*p* value
**Clinical information**			
Male (*n*%)	60	54.3	> 0.9999
Age (years) ^a^	2.5	2	-
Body weight (kg) ^a^	4.2	3.6	-
Open mouth breathing (*n*%)	-	64.29	-
Paresis (*n*%)	-	57.14	-
**Coagulation markers**			
D-dimer (ng/ml) ^a^	208.54 ± 10.92**	372.19 ± 58.28**	0.0082
Prothrombin time (seconds) ^a^	11.08 ± 0.23****	9.8 ± 0.15****	< 0.0001
***MYBPC3*** **gene mutation**			
A31P Mutation rate (*n*%)	-	-	-
A74T Mutation rate (*n*%)	-	57.14	-
Heterozygous	-	57.14	-
Homozygous	-	-	-

*MYBPC3* = myosin-binding protein C3, ^a^ = mean ± standard error of mean (SEM)

The statistically significant difference was performed using chi’s square and t-test in GraphPad Prism. ** *p* < 0. 01, ****p < 0.0001 were considered statistically significant

### Coagulation profile

**Fig. 1:**
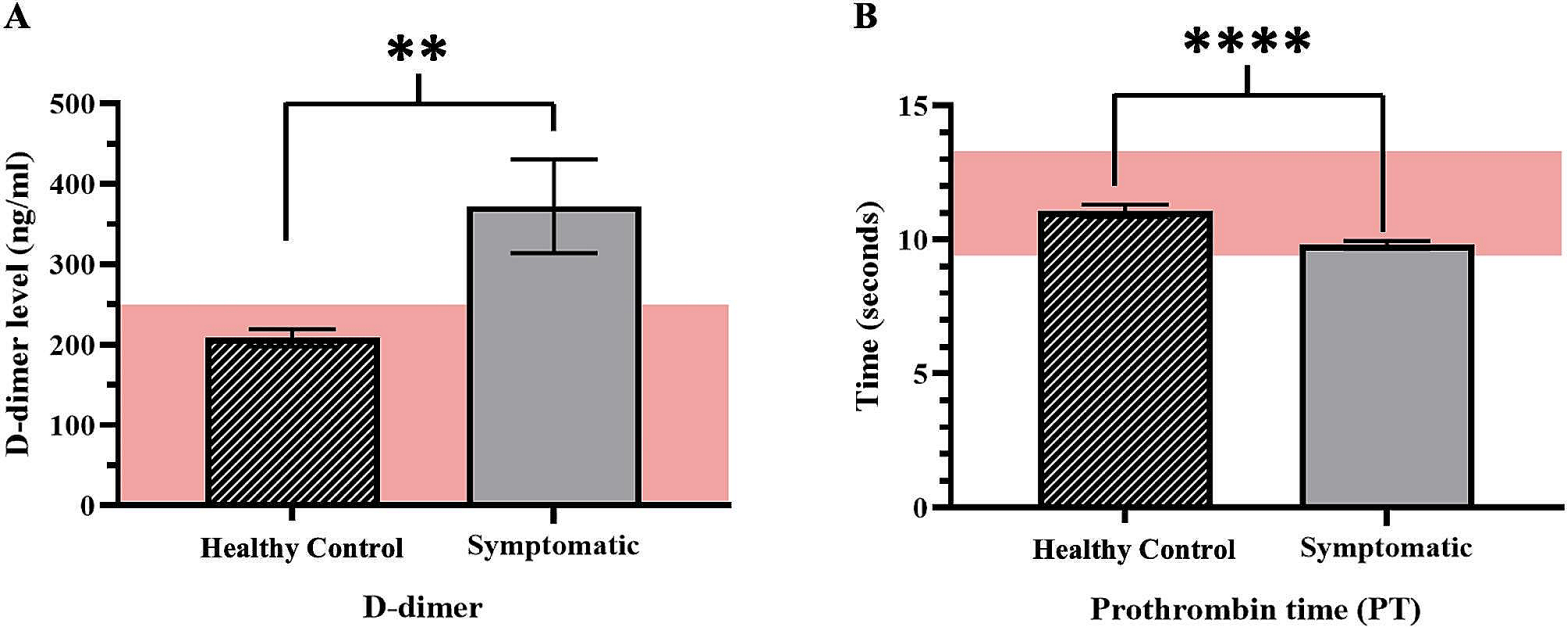
The results of coagulation markers, including **(A)** D-dimer level and **(B)** Prothrombin time (PT). All data was analyzed by t-test. The mean ± standard error of the mean (SEM) of coagulation markers levels of healthy and symptomatic HCM is illustrated. The area behind both bar graphs showed the standard ranges of each coagulation marker. ** *p* < 0. 01, ****p < 0.0001 were considered statistically significant

Our coagulation marker findings indicated that the D-dimer levels in healthy and symptomatic HCM cats were 208.54 ± 10.92 and 372.19 ± 58.28 ng/ml, respectively; the level of D-dimer was significantly elevated in symptomatic cats compared to healthy cats (*p* = 0.0082). Additionally, PT was decreased considerably in symptomatic cats compared to healthy cats (*p* < 0.0001); however, PT values in healthy and symptomatic cats were within normal limits (11.08 ± 0.23 and 9.8 ± 0.15 s, respectively). The coagulation profile results are illustrated in Tables 1 and 2, and Fig. 1.

**Table 2:** Echocardiographic results in enrolled cats

Echocardiographic Parameters	Healthy Control cats (*n* = 15)	Symptomatic cats (*n* = 14)	*p* value
IVSd (cm)	0.47 ± 0.10**	0.61 ± 0.12**	0.002
IVSs (cm)	0.60 ± 0.13**	0.72 ± 0.13**	0.0155
LVPWd (cm)	0.47 ± 0.07***	0.61 ± 0.13***	0.0011
LVPWs (cm)	0.58 ± 0.12*	0.69 ± 0.12*	0.0203
LVIDd (cm)	1.50 ± 0.23	2.00 ± 2.25	0.3990
LVIDs (cm)	0.87 ± 0.27	0.77 ± 0.25	0.3109
FS (%)	42.98 ± 11.20	46.54 ± 9.48	0.3655
LA diameter (cm)	0.99 ± 0.19**	1.24 ± 0.23**	0.0035
AO diameter (cm)	0.72 ± 0.12	0.72 ± 0.09	1.0000
LA/AO ratio	1.40 ± 0.15**	1.76 ± 0.41**	0.0036
MV E/A ratio	1.00 ± 0.10	0.96 ± 0.31	0.6389
IVRT (seconds)	0.042 ± 0.006****	0.065 ± 0.02****	0.0002

IVSd = interventricular septal thickness at end-diastole, IVSs = interventricular septal thickness at end-systole, LVPWd = left ventricular proximal wall diameter at end-diastole, LVPWs = left ventricular proximal wall diameter at end-systole, LVIDd = left ventricular internal diameter at end-diastole, LVIDs = left ventricular internal diameter at end-systole, FS = fractional shortening, LA = left atrium, AO = aorta, MV E/A ratio = ratio of transmitral E and A peak velocity, IVRT = isovolumic relaxation time

The statistically significant difference was performed using a paired t-test. * *p* < 0.05, ** *p* < 0.01, *** *p* < 0.0005, ***** *p* < 0.0001 were considered statistically significant. The values were demonstrated in mean ± standard deviation (SD).

### Echocardiography

All recruited cats were evaluated for cardiac morphology and function using echocardiography. The echocardiographic results revealed that the diameters of the IVSd, IVSs, LVPWd, LVPWs, LA, and the LA/AO ratio were significantly increased in symptomatic HCM cats. Our study determined diastolic function by measuring isovolumic relaxation time (IVRT), as in Table 3. IVRT was prolonged considerably in symptomatic HCM cats. This result indicated that symptomatic HCM cats had impaired diastolic function.

**Table 3:** Clinical signs and echocardiographic staging in enrolled cats

Enrolled cats (*n* = 29)	Healthy control cats (*n* = 15)	Symptomatic cats (*n*-14)
**Physical examination**		
Open mouth breathing	-	9
Paresis	-	8
Open mouth breathing and paresis	-	3
**Echocardiographic staging**		
Stage A	15	-
Stage B1	-	5
Stage B2	-	4
Stage C	-	5

### Proteomics analysis using MALDI-TOF and LC-MS/MS

**Fig. 2:**
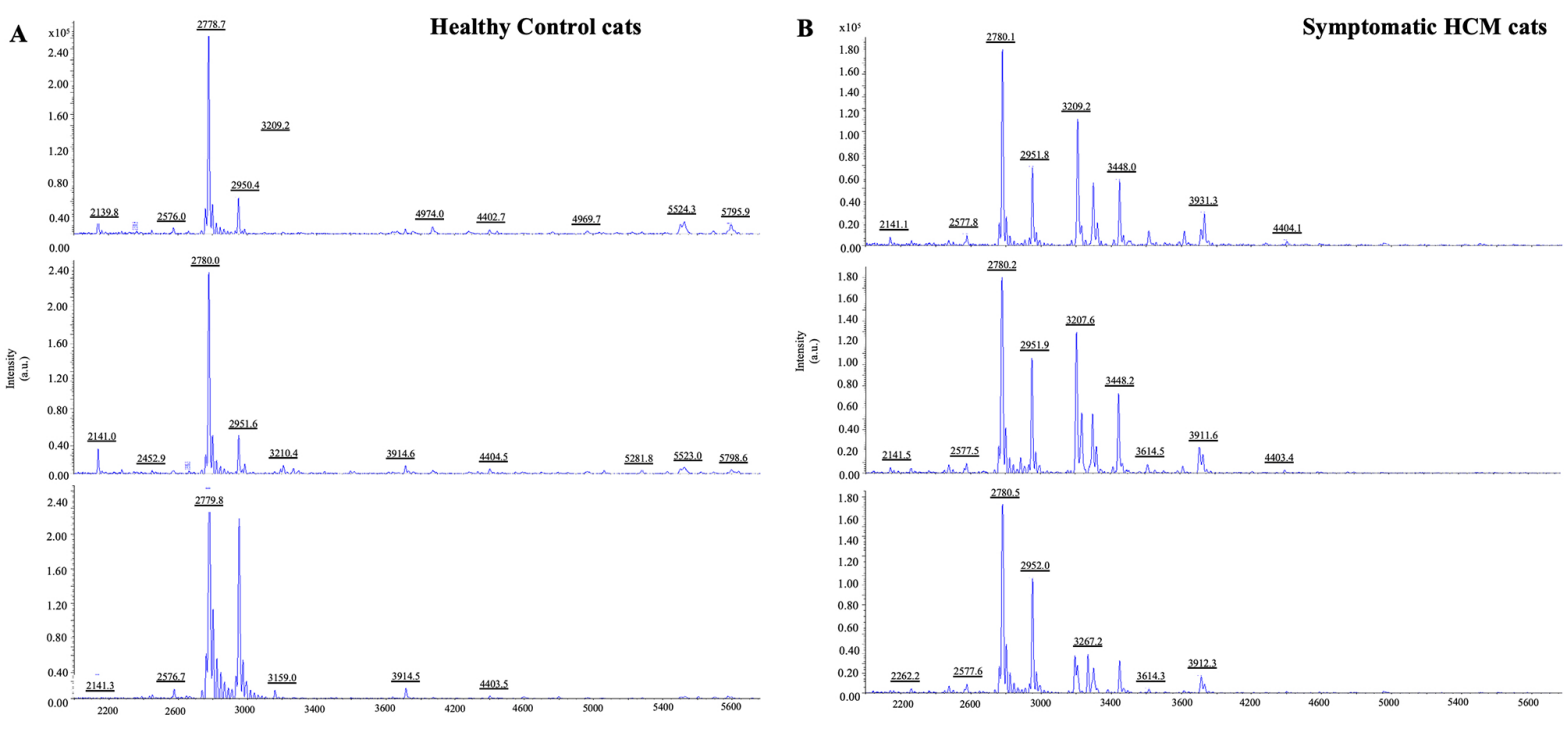
Peptide mass fingerprints (PMFs) from MALDI-TOF mass spectrometry with **(A)** healthy control cats and **(B)** Symptomatic HCM cats

**Fig. 3:**
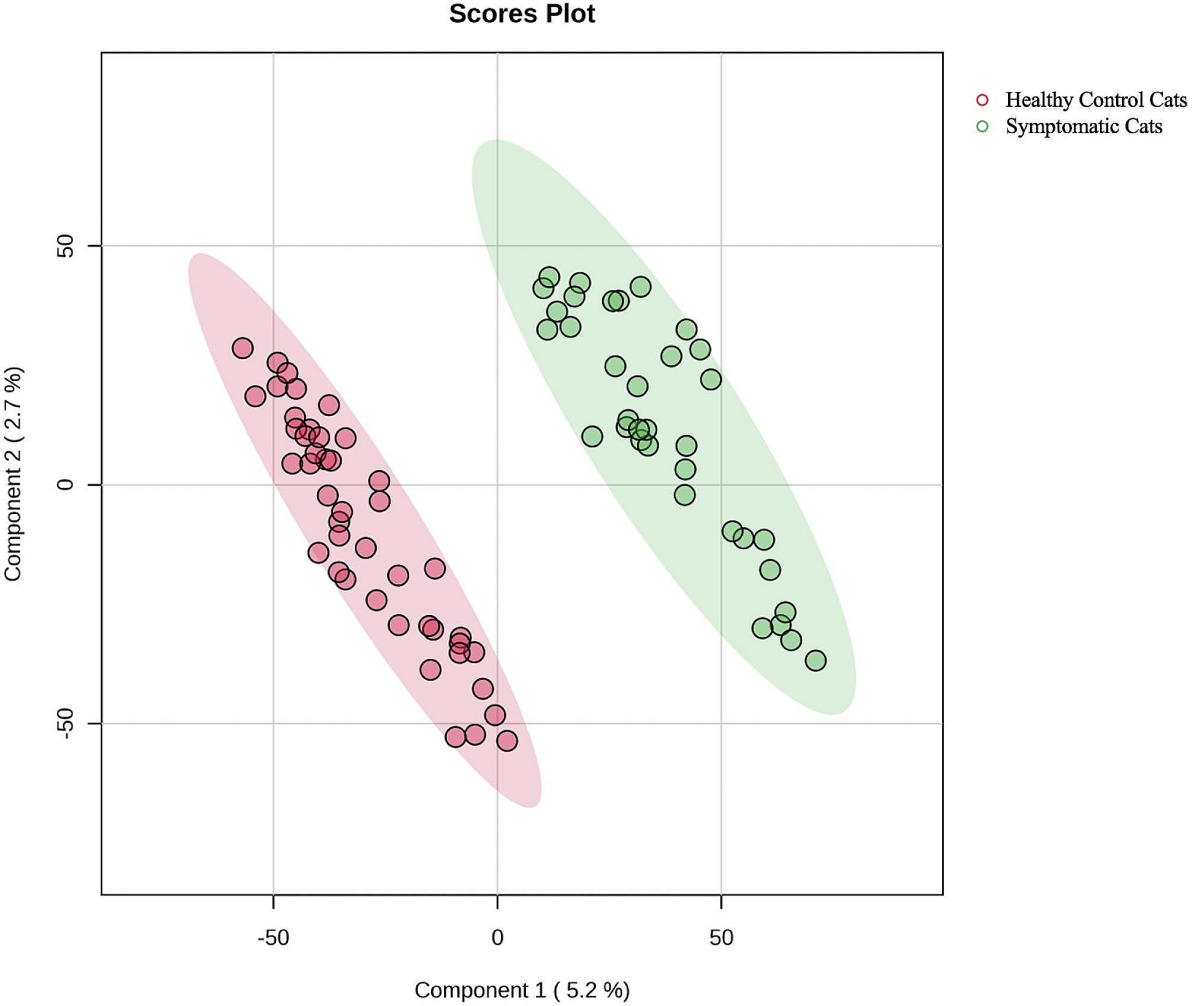
Partial least squares discriminant analysis (PLS-DA) score plot of the proteins in healthy cats (red) and symptomatic HCM (green) demonstrated the protein expression in both groups that completely separated

Twenty-nine serums from HCM cats with healthy and symptomatic were analyzed with MALDI-TOF and LC-MS/MS. The results of MALDI-TOF mass spectrometry revealed the different mass peaks of peptide mass fingerprints (PMFs) from 2 groups of healthy control cats (Fig. 2A). and symptomatic HCM cats (Fig. 2B). Moreover, serum protein expression in cats with symptomatic and healthy was elucidated by LC-MS/MS. It was revealed that 269 proteins were differentially expressed between healthy and symptomatic HCM cats. The cluster analysis results were represented with a partial least squares discriminant analysis (PLS-DA) demonstrating the completely distinguished proteins from two analytical groups (Fig. 3).

**Fig. 4:**
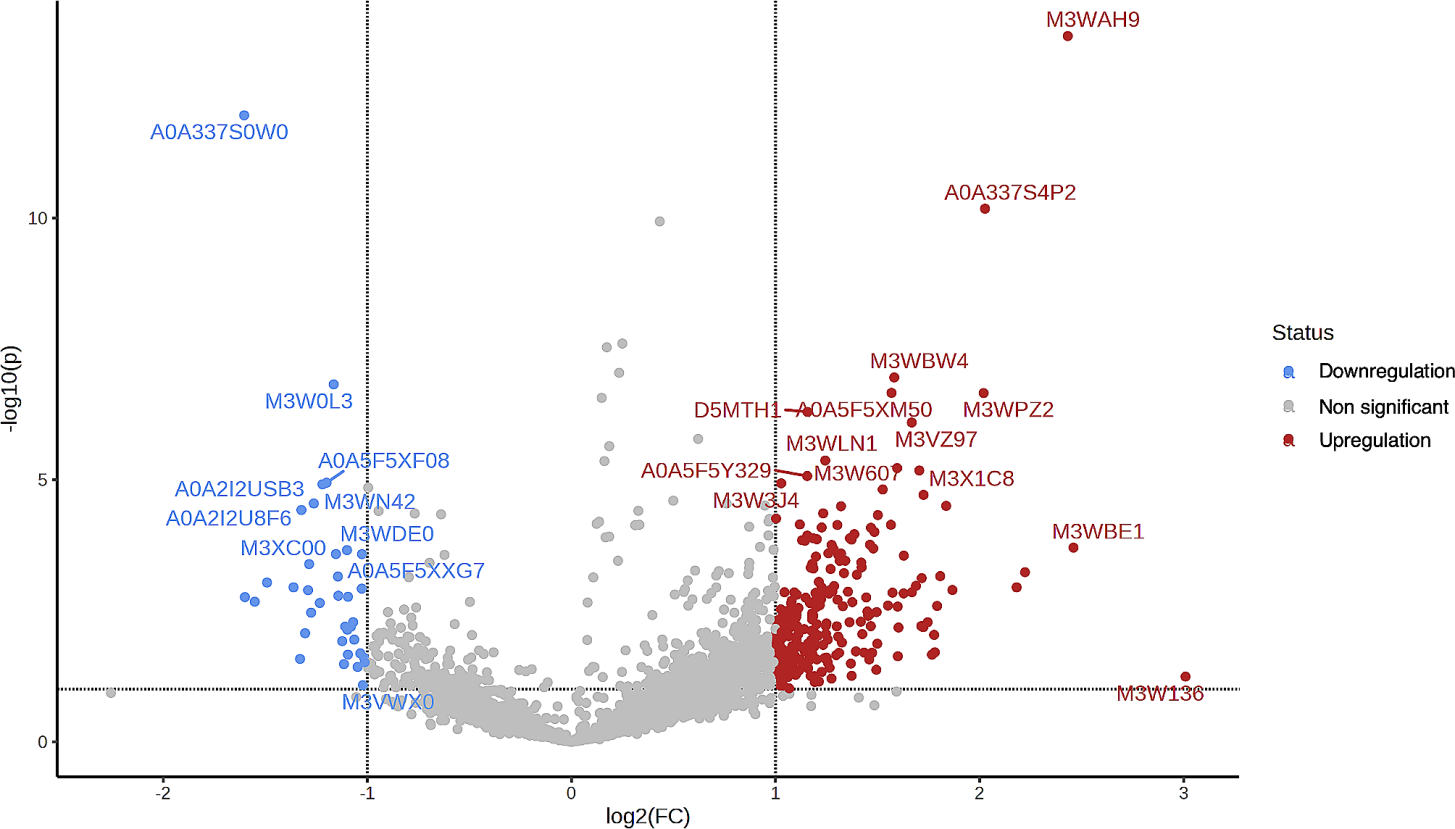
Volcano plots representing the results of comparisons between healthy control cats and symptomatic HCM cats, including downregulation (blue), non-significant (gray), and upregulation (red)

The results revealed that the expression of 40 proteins was upregulated in symptomatic HCM cats, while that of 229 proteins was downregulated. Integrin subunit alpha M (ITGAM), elongin B (ELOB), and fibrillin 2 (FBN2) were upregulated, and zinc finger protein 316 (ZNF316), a regulatory protein for the transcription of RNA polymerase II and ectonucleoside triphosphate diphosphohydrolase 8 (ENTPD8), a regulatory protein for controlling profibrotic nucleotide, were down-regulated. Moreover, the ALMS1 centrosome and basal body-associated protein encoded by the *ALMS1* gene were downregulated in symptomatic HCM cats. The figure of the volcano plot is illustrated in Fig. 4.

**Fig. 5:**
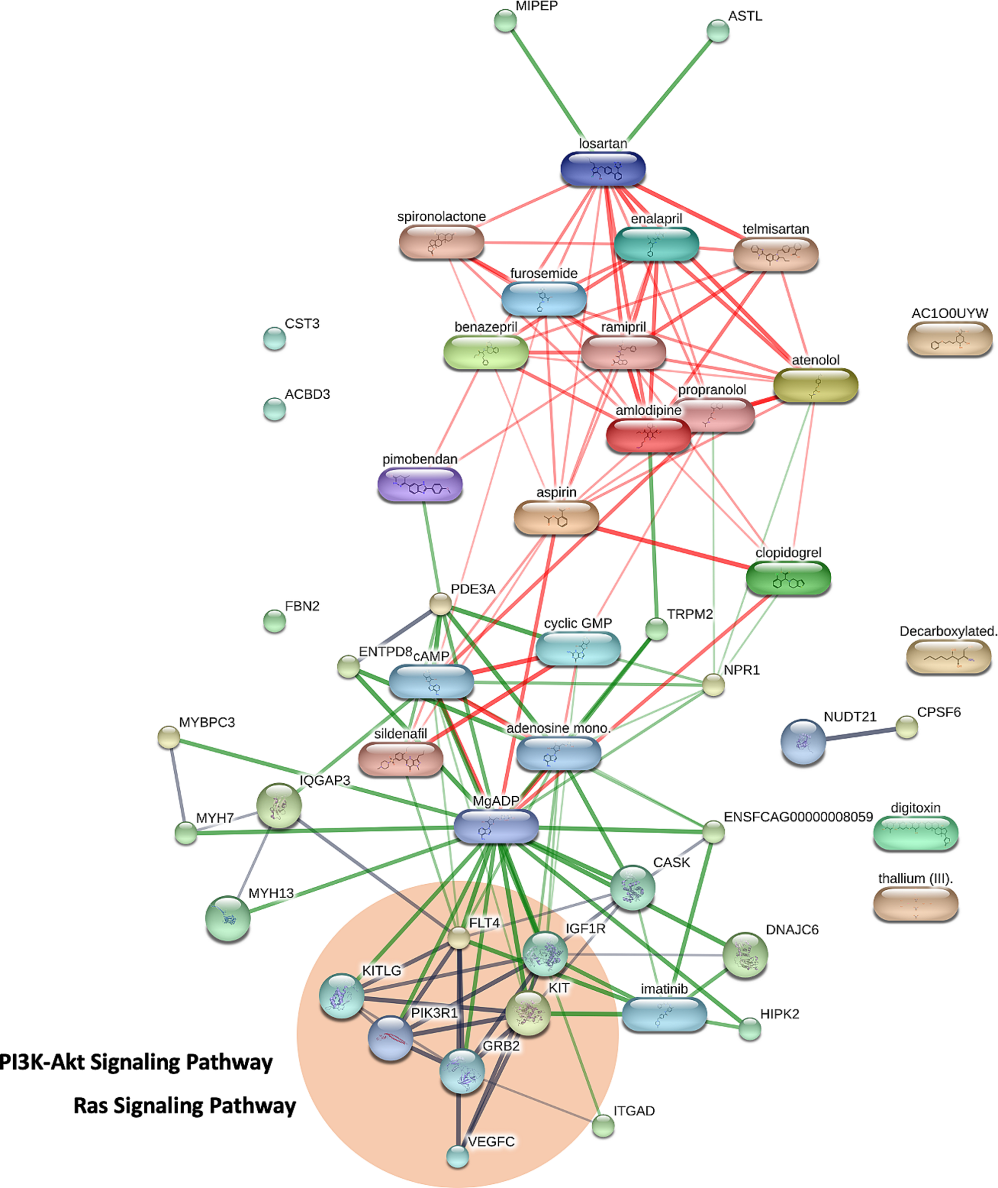
The protein and drug interaction networks form LC-MS/MS. The circle indicates the protein associated with pathways for cardiac hypertrophy

The protein and cardiovascular drug interactions were determined using the Stitch version 5.0 software. Pimobendan, the positive inotropic medication, was associated with phosphodiesterase 3 A (PDE3A) and ENTPD8 protein downregulation in cats with symptomatic HCM. Moreover, guanylate cyclase (NPR1) was associated with clopidogrel, a crucial therapeutic agent for antiplatelet control of arterial thrombosis in cats, and beta-blocker agents (propranolol and atenolol) for controlling heart rate and reducing blood pressure. Additionally, a nonselective calcium-permeable cation channel, transient receptor potential cation channel subfamily M member 2 (TRPM2), was found to have a potential relationship with amlodipine, an antihypertensive drug. Losartan, an angiotensin receptor blocker (ARB), was related to mitochondrial intermediate peptidase (MIPEP) and astacin-like metalloendopeptidase (ASTL). In addition, protein and drug interactions were indicated in the pathways associated with cardiac pathologies, such as the Ras signaling pathway and PI3K-Akt signaling pathway. The identified proteins correlated with these pathways included fms-related receptor tyrosine kinase 4 (FLT4) and tyrosine-protein kinase receptor (IGF1R), as shown in Fig. 5 in the circle.

## Discussion

In this study, we determined the differences in the coagulation markers D-dimer and PT in cats with healthy and symptomatic HCM. Serum proteomics analysis was also performed with LC-tandem mass spectrometry for the potentially massive protein evaluation.

Dilation of the LA was observed in cats with symptomatic HCM. Previous studies demonstrated that severe LA enlargement affected the diminishing of contractility by decreasing the percentage of LA fractional shortening. Consequently, stasis of the blood and thrombosis in the LV chamber occurred. These situations led to ATE due to clinical HCM [22]. Currently, multiple coagulation markers affect the hypercoagulation stage in cats. Our findings revealed that cats with symptomatic HCM had elevated D-dimer levels. However, PT and D-dimer levels in our study do not strongly support the presence of hypercoagulation. In human medicine, serum D-dimer was used as a cut-off value with high sensitivity, and specificity for screening myocardial infarction and for predicting pulmonary embolism [23, 24]. However, the use of the D-dimer as a cut-off value for HCM in cats requires further study.

Moreover, the D-dimer concentration in symptomatic HCM cats exceeded the average value. PT is one of the parameters for the evaluation of coagulability in cats. In our study, PT was increased in symptomatic HCM cats. However, PT in both healthy and symptomatic cats was within the normal range. Previous studies agree that hypercoagulability is highly common in cats with cardiomyopathy, identified by spontaneous echo contrast (SEC) in the LA chamber and ATE [25].In a previous study, it has been revealed that cats with HCM, ATE, and SEC had increased D-dimer concentrations [26]. A report on asymptomatic HCM cats revealed that approximately 16% had hypercoagulability due to the elevation of D-dimer levels. However, that study did not alter PT in asymptomatic HCM [27]. In human medicine, increasing D-dimer concentrations were notably exhibited in the older population [28].

In this study, the age of the recruited cats was similar to that of young adults. This result indicated that age was not a confounding factor affecting this experiment. Therefore, D-dimer proved beneficial for detecting and monitoring HCM in cats to prevent the worst clinical manifestation of ATE.

In the present study, the E/A ratio in the symptomatic group was not different from that in the control group, and IVRT was significantly different among groups. The results of this study are similar to a previous study [29] which reported that left atrial enlargement was considered a morphophysiological expression of LV diastolic dysfunction, with increasing left atrial size corresponding to progressively worse LV diastolic function and atrial hypertension. The E/A ratio was a useful parameter in detecting congestive heart failure in cats with HCM, and several Doppler echocardiography variables have been assessed to evaluate the left ventricular filling pressure such as the ratio of transmitral flow velocity between peak E and peak (E/A), and the ratio of E velocity to the relaxation time (IVRT).The upregulation of integrin subunit alpha M (ITGAM), elongin B (ELOB), and fibrillin 2 (FBN2) was observed in this study. It has been documented that ITGAM is potentially related to cardiac hypertrophy. Additionally, previous studies found that the long noncoding RNA Pvt1 regulates TNF/Met/ITGAM/Bst1 expression, impacting myocardial inflammation, hypertrophy, apoptosis, and contractility [30]. The utility of ITGAM suggests that decreasing ITGAM expression may ameliorate myocardial infarction [31] and that ITGAM gene polymorphism is a beneficial marker for determining cardiac cachexia in people with chronic congestive heart failure [32]. Elongin B (ELOB), a ubiquitin-like protein, is responsible for the activation of elongation of RNA polymerase [33]. The expression of ELOB is induced by spermatogenesis via RNA maturation. Currently, information on ELOB expression in cardiac diseases is limited [34]. However, the ubiquitin-proteasome system associated with ELOB is the protein degradation system that controls deleterious proteins in the cell [35]. Fibrillin 2 (FBN2) is an extracellular glycoprotein. This protein is responsible for the components of elastic fibers and cell adhesion in most tissues [36]. It has been reported that mutation of the *FBN2* gene is the cause of Beals-Hecht syndrome, a congenital disease of connective tissue [37]. Furthermore, a few reports have demonstrated that FBN2 in the ascending aorta is overexpressed in congenital heart disease of the bicuspid aortic valve (BAV) [38].

Zinc finger protein 316 (ZNF316) and ectonucleoside triphosphate diphosphohydrolase 8 (ENTPD8) exhibited decreased expression in symptomatic HCM cats. ZNF316 belongs to the zinc finger protein family. Zinc finger proteins are transcription factors associated with the Krüppel-like factor (KLF) family. Previous reports have established that zinc finger protein 580 (ZFP580) can inhibit cardiac hypertrophy in H9c2 cells by preventing hypoxia-induced apoptosis through the TGF-β1/Smad signaling pathway [39, 40]. Therefore, the reduced expression of ZNF316 in symptomatic HCM cats may contribute to cardiac hypertrophy and represent a novel therapy target in managing feline HCM. A previous study reported that cellular homeostasis and fibrotic response involve the integration of signaling that is pro-fibrotic by ATP and anti-fibrotic by adenosine and that is regulated by ENTPDs. Moreover, ENTPD protein was associated with cardiac fibrosis and myocardial ischemic injury [41]. Ectonucleoside triphosphate diphosphohydrolase 8 (ENTPD8) was also downregulated in this present study. It has been documented that decreasing ENTPD8 in renal epithelial cells and liver parenchyma induces profibrotic signaling via the TGF-β1 pathway [42, 43]. Consequently, the suppression of ENTPD8 expression may be linked to feline HCM pathogenesis. In addition, the protein-drug interaction analysis showed that ENTPD8 was related to phosphodiesterase 3 A (PDE3A), downregulated in this study. ALMS1, the protein encoded by the *ALMS1* gene, causes Alström syndrome through *ALMS1* gene polymorphisms [44, 45]. In Sphynx cats, it has been revealed that the mutation of the *ALMS1* gene is associated with the development of cardiac hypertrophy [46, 47]. However, the ALMS1 gene mutation status was limited in our study. Hence, the evaluation of *ALMS1* gene mutation status may require further elucidation.

Proteomic analysis revealed that FLT4 and IGF1R are proteins associated with the Ras signaling pathway and the PI3K-Akt signaling pathway. FLT4 is involved in the development of the cardiovascular system in the embryonic stage, the development of the vascular network, and lymphangiogenesis [48]. Moreover, the *FLT4* gene encodes the vascular endothelial growth factor C (VEGFC) protein, associated with angiogenesis and endothelial cell growth [49]. It has been reported that the mutations of the *FLT* gene are deleterious and related to congenital diseases such as tetralogy of Fallot [50, 51]. The mechanism of the IGF1R-AKT-VEGF pathway is stimulated according to IGF1R expression [52]. Activation of the EGFR/IGF1R signaling pathway is implicated in impaired relaxation.

Furthermore, various publications have established that the Ras and PI3K-Akt signaling pathways contribute to cardiac hypertrophy and fibrosis [53–56]. Our proteomics results are similar to the study that reported that the plasma proteomic Ras-MAPK pathway was involved in HCM in heart failure patients [57]. Consequently, the Ras signaling pathway and PI3K-Akt signaling pathway may be associated with symptomatic HCM development and possible targets for novel cat therapeutic approaches.

This present study intends to compare coagulation markers and to determine differences in proteomics profiles between healthy and symptomatic HCM in cats. There are many differences in protein expression between clinical and healthy cats that could provide a potential biomarker. However, a large study group with a homogeneous population would likely facilitate the use of these data in clinical use, and this is a limitation of our study.

Another limitation is that this present study was a cross-sectional study using convenience sampling. Therefore, in this study, a comprehensive examination of three important coagulation parameters, such as fibrinogen, fibrinogen degradation products, and D-dimer, was not available. In further studies, the relationship among coagulation parameters needs to be further investigated. However, previous studies suggested that the D-dimer was suggested to be an independent predictor for good functional outcomes for human patients receiving intravenous thrombolysis therapy [58].

In conclusion, elevated D-dimer levels and decreased PT could serve as beneficial indicators for the prediction of HCM in cats. Proteomic analysis identified critical proteins that are dysregulated in cats with HCM. Furthermore, the Ras and PI3K-Akt pathways may be promising targets for therapeutic intervention in feline HCM. However, the use of D-dimer as a cut-off value for the detection of HCM and a better understanding of disease mechanisms for novel therapeutic approaches are still challenging in cats.

## Data Availability

No datasets were generated or analysed during the current study.

## Abbreviations

ASTL: Astacin-like metalloendopeptidase
ATE: Arterial thromboembolism
DIC: Disseminated intravascular coagulation
ELOB: Elongin B
ENTPD8: Ectonucleoside triphosphate diphosphohydrolase 8
FBN2: Fibrillin 2
FLT4: Fms-related receptor tyrosine kinase 4
FS: Fractional shortening
HCM: Hypertrophic cardiomyopathy
IGF1R: Tyrosine-protein kinase receptor
ITGAM: Integrin subunit alpha M, isovolumic relaxation time
IVSd: Interventricular septal thickness at end-diastole
IVSs: Interventricular septal thickness at end-systole
LA/AO: ratio of left atrium and aorta
LC-MS/MS: Liquid chromatography-tandem mass spectrometry
LVIDd: Left ventricular internal diameter at end-diastole
LVIDs: Left ventricular internal diameter at end-systole
LVPWd: Left ventricular proximal wall diameter at end-diastole
LVPWs: Left ventricular proximal wall diameter at end-systole
MALDI-TOF: Matrix-assisted laser desorption ionization time-of-flight
MIPEP: Mitochondrial intermediate peptidase
MV E/A ratio: ratio of transmitral E and A peak velocity
MYBPC3: Myosin-binding protein C3 mutation
NPR1: Guanylate cyclase
PDE3A: Phosphodiesterase 3 A
PT: Prothrombin time
SEC: Spontaneous echo contrast
VEGFC: Vascular endothelial growth factor C
ZNF316: Zinc finger protein 316
